# Macromonomers as a Novel Way to Investigate and Tailor Silicon-Oxycarbide-Based Materials Obtained from Polymeric Preceramic Precursors

**DOI:** 10.3390/ma14133703

**Published:** 2021-07-02

**Authors:** Maria Owińska, Paulina Matoga, Piotr Jeleń, Elżbieta Długoń, Bartosz Handke, Wiktor Niemiec

**Affiliations:** Faculty of Materials Science and Ceramics, AGH-University of Science and Technology, Al. Mickiewicza 30, 30-059 Kraków, Poland; owinska@agh.edu.pl (M.O.); paulina.matoga@interia.pl (P.M.); pjelen@agh.edu.pl (P.J.); dlugon@agh.edu.pl (E.D.); bhandke@agh.edu.pl (B.H.)

**Keywords:** silicon oxycarbide, black glass, macromonomer, sol-gel, preceramic polymer, IR, TG

## Abstract

It has been shown that bifunctional monomers (D units), which are used to increase the carbon content in silicon oxycarbide precursors, can form volatile oligomers, thus affecting the amount of carbon available during the transition into the final material in the annealing process. Additionally, an uneven distribution of carbon-rich mers may lead to the formation of a free-carbon phase, instead of the incorporation of carbon atoms into the silicon matrix. In this study, a novel two-step approach was utilized. Firstly, a macromonomer containing a number of structural units with precise structure was synthesized, which was later polycondensed into a ceramic precursor. Chlorodimethylsilane modified 2,4,6,8-tetramethylcyclotetrasiloxane was used as a silicon oxycarbide precursor monomer containing both T and D structural units (i.e., silicon atoms bonded to three and two oxygen atoms, respectively), with well-defined interconnections between structural units. Such a macromonomer prevents the formation of small siloxane rings, and has a very limited number of possible combinations of structural units neighboring each silicon atom. This, after investigation using IR, XRD, TG and elemental analysis, gave insight into the effect of “anchoring” silicon atoms bonded to two methyl groups, as well as the impact of their distribution in comparison to the materials obtained using simple monomers containing a single silicon atom (structural unit).

## 1. Introduction

Silicon-oxycarbide-based black glass is a composite material whose possible uses are investigated in a wide range of applications, due to its composite structure. Silicon and oxygen atoms can only be found in the bulk of the material, where they form the glassy phase similar to silica glass. Carbon atoms on the other hand can be found both in the bulk of the material—where they replace pairs of oxygen atoms, increasing the interconnectivity of the bulk matrix—and in the form of a free-carbon phase. This phase can take two main forms: turbostratic carbon domains inside the ceramic bulk, or graphene-like sheets intersecting the whole material [1]. Usually, the creation of the latter type is caused by annealing in temperatures exceeding 1100 °C [1]. Such a structure has a number of advantageous effects on material properties. The increased bond density of the bulk and composite structure enhances the material’s mechanical resistance [2,3], as well as its thermal and chemical resistance [4,5]. An additional effect of the carbon phase (especially the graphene-like type) is increased conductivity. As both the free-carbon content and its type can vary, silicon-oxycarbide-based black glass can be a semiconductor or insulator, and the addition of carbon forms, for example, can make the material conductive [6]. Such properties make silicon oxycarbide a promising material for protective coatings [7,8], electrodes in batteries [9], fuel cells [10], catalysis [11,12], or in medicine [13,14].

The synthesis of silicon-oxycarbide-based black glass is impossible with standard powder methods. The two main routes are either atom-deposition-based [15,16,17] or organosilicon-polymeric-preceramic-precursor-based. In the latter, either commercially available polysiloxanes [18,19], networked organosilicon polymers [20,21], or polysilsesquioxane gels obtained using the sol-gel method [22,23] can be annealed at temperatures exceeding 700 °C [24]. The last route is especially interesting for scientists, as it allows the easy customization of the preceramic polymer atom content by mixing small alkoxysilanes with a variable number of Si-C bonds [25], as well as different organic side groups [26]. The problem is that monomers with two or three Si-C bonds have a greater tendency to form volatile oligomers that can evaporate before the glass transition, thus effectively lowering the carbon content in the preceramic material [27]. This, as well as volatile organic compound formation in the ceramization process, leads to an interesting effect whereby even great differences in carbon content in the preceramic precursor can lead to a similar carbon content in the resulting silicon-oxycarbide-based black glass, although these materials have different properties [25].

**Figure 1:**
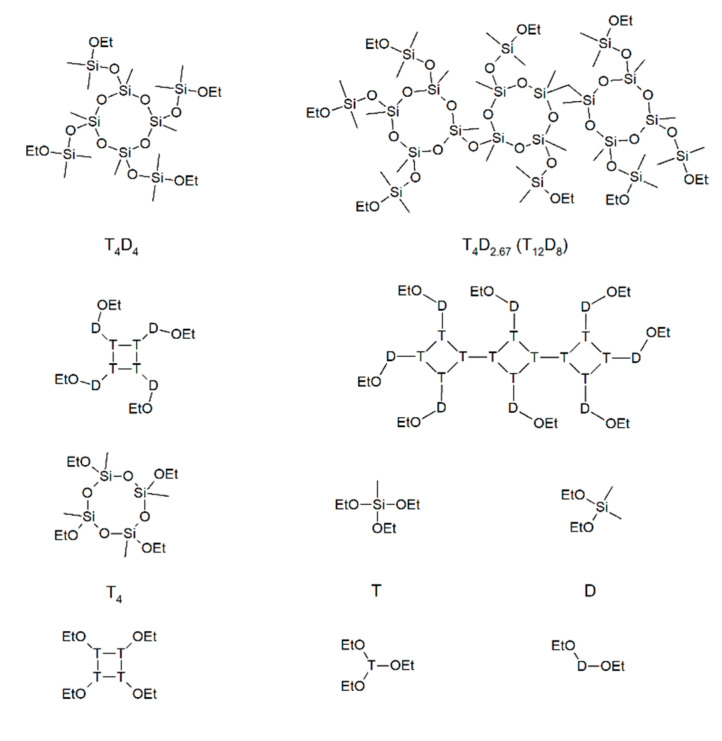
Molecular formulas (denoted at the top) and structural unit compositions (depicted below each molecular formula) of the target macromonomer (T_4_D_4_), one of the isomers of the obtained macromonomer (T_4_D_2.67_), and other monomers used—2,4,6,8-tetraethoxy-2,4,6,8-tetramethylcyclotetrasiloxane (T_4_), diethoxydimethylsilane (D), and triethoxymethylsilane (T).

In this work, 2,4,6,8-tetramethylcyclotetrasiloxane (D_4_^H^) was modified with chlorodimethylsilane to obtain a monomer containing both T structural units (i.e., silicon atom bonded with three oxygen atoms and one carbon atom) and D structural units (i.e., silicon atoms bonded with two oxygen atoms and two carbon atoms). Such a robust monomer would prevent the formation of carbon-rich oligomers, thus preventing carbon loss in the oligomer evaporation step of the annealing process. In addition, a number of precursors with a varying silicon to carbon atom ratio using either the mixtures of the aforementioned robust monomer and 2,4,6,8-tetraethoxy-2,4,6,8-tetramethylcyclotetrasiloxane or diethoxydimethylsilane and triethoxymethylsilane as reference samples were prepared and annealed to investigate the impact of the monomer size on the obtained silicon-oxycarbide-based black glass. Both the molecular formulas and structural unit compositions of the monomers are shown in Figure 1. Even though mixtures of simple monomers are well described in the literature [25], the reference samples were prepared and investigated because the synthesis method required for a robust monomer gel formation affects the polycondensation process, which can lead to differences in both the structure and properties of the final material [28].

## 2. Materials and Methods

### 2.1. Synthesis of Macromonomer (T_4_D_2.67_)

The macromonomer was obtained through a three-step synthesis based on the procedures described in the literature [29,30]. At the first stage, 2,4,6,8-tetrahydroxyl-2,4,6,8-tetramethylcyclotetrasiloxane (T_4_^OH^) was obtained through the hydrolysis of 2,4,6,8-tetrahydro-2,4,6,8-tetramethylcyclotetrasiloxane (D_4_^H^). The reaction of the T_4_^OH^ with chlorodimethylsilane (Me_2_SiHCl) in toluene solution at room temperature, in the presence of pyridine as an acceptor of HCl, gives the hydride T_4_M_2.67_^H^. The last step of the procedure was the substitution of hydride atoms with etoxyl groups in T_4_M_2.67_^H^, and was carried out immediately prior to hydrolytic polycondensation of the obtained macromonomer.

#### 2.1.1. Materials

2,4,6,8-tetrahydro-2,4,6,8-tetramethylcyclotetrasiloxane (D_4_^H^) was purchased from ABCR (Karlsruhe, Germany) and used as supplied. Tetrahydrofuran (THF) and toluene were supplied by Avantor Performance Materials (Gliwice, Poland) and were distilled from sodium-benzophenone prior to use. Pyridine was supplied by ChemPur (Piekary Śląskie, Poland) and distilled from calcium hydride before use. Pd/C (5% activated), Celite^®^, chlorodimethylsilane (98%) (Me_2_SiHCl) were supplied by Sigma-Aldrich (St. Louis, MO, USA). All other materials (anhydrous magnesium sulphate, calcium hydride) were purchased from Avantor Performance Materials.

#### 2.1.2. Synthesis of 2,4,6,8-Tetrahydroxyl-2,4,6,8-Tetramethylcyclotetrasiloxane (T_4_^OH^)

In a 250 mL round-bottomed flask, hydrolysis of the D_4_^H^ (10 mL, 0.042 mol) was carried out using distilled water (3.4 mL, 0.189 mol) and 5% activated Pd/C (0.86 g) for 2 h in THF (116 mL) at 10 °C. After the reaction, Pd/C was removed using Teflon syringe filters with a pore size of 250 nm, and then the solution was dried over anhydrous magnesium sulphate and Celite^®^, followed by filtering and slowly evaporating excess THF at temperatures below 20 °C. The product, T_4_^OH^ (12.64 g, yield = 99.8%), was stored as solidifying oil at 15 °C prior to use. Due to trace amounts of water left behind, the highly reactive silanol groups underwent condensation, which resulted in oligomer formation.

#### 2.1.3. Synthesis of Chlorodimethylsilane Modified 2,4,6,8-Tetrahydroxyl-2,4,6,8-Tetramethylcyclotetrasiloxane (T_4_M_2.67_^H^)

Toluene (100 mL), pyridine (20.1 mL, 0.250 mol) and chlorodimethylsilane (30 mL, 0.250 mol) were charged in a three-neck flask equipped with a stirrer and reflux condenser with a calcium chloride tube. The mixture was stirred at room temperature for 10 min, and then the solution of T_4_^OH^ (12.64 g, 0.041 mol) in dry toluene (50 mL) and THF (60 mL) were slowly added using a dropping funnel. The reaction was conducted at room temperature for 6 h. The solid precipitate was filtered off, and the filtrate was washed with water three times. The combined organic layers were dried over anhydrous magnesium sulfate, followed by evaporation of the solvents in vacuo to give T_4_M_2.67_^H^ as a colorless oil (17.1 g, yield = 80%). The obtained compound was used in the next step of the synthesis without further purification.

### 2.2. Synthesis of Preceramic Polymeric Precursors

All precursor syntheses were based on a procedure described in our previous article [28]. Triethoxymethylsilane (98%, Acros Organics) and diethoxydimethylsilane (97%, Fluka) were used for the preparation of reference samples, while 2,4,6,8-tetramethylcyclotetrasiloxane (95%, ABCR) was used as a source of additional T structural units (T_4_) in the case of mixtures containing the synthesized macromonomer. The preceramic precursors with a T to D structural unit ratio of 1:1 (sample T:D), 3:2 (samples T_4_D_2.67_ and 3T:2D), 2:1 (samples 3T_4_D_2.67_:T_4_ and 3T:2D) and 4:1 (samples 3T_4_D_2.67_:5T_4_ and 4T:D) were prepared. The sample names include the monomers used and their molar ratio. The general procedure of all syntheses started with the mixing of monomers to obtain the correct ratio of structural units (with the exception of 3:2 ratio prepared using large monomers, as it was obtained from T_4_M_2.67_^H^ without any additions), and then stirring the mixture for 15 min. In the case of large monomers, the Si-H bonds were substituted for ethoxy groups. A suspension of 1% monomer mass of Pd/C (5% Pd, Sigma Aldrich) and one mole of ethanol per mole of silicon atoms and Si-H bonds in the precursor were added dropwise to the precursor and stirred for 2 h, which led to the substitution of all hydrogen atoms with reactive groups [28]. The catalyst was then removed using Teflon syringe filters with a pore size of 250 nm. The monomers where then placed in a closed flask equipped with a gas washing bottle filled with silicon oil to keep constant air pressure, with air substituted for dry argon. Then, in the case of monomers containing more than one structural unit, the same volume of tetrahydrofuran (P.A., Avantor Performance Materials) as the volume of monomer mixture was added and stirred for 15 min to prevent phase separation after the addition of a water-based catalyst. As our aforementioned studies have shown that such addition does not affect precursor structure in the case of single structural unit monomers, this stage was omitted in their case. Then a water-based catalyst containing 2 moles of twice distilled water for each mole of silicon atoms in the monomers mixture with hydrochloric acid (35–38%, P.A., Avantor Performance Materials) in the amount needed to achieve pH 4.5 and 1 mole of ethanol per mole of silicon atoms in the monomers mixture was added dropwise. The reagents were stirred for 4 h in the case of simple monomers and for 48 h in the case of complex monomers, and then were poured on Petri dishes (3 mm mixture height level). Then they were placed in an exiccator with dry silica gel until a gel was formed, and then kept at 70 °C for 2 weeks for further solvent removal. Precursors based on the T_4_D_2.67_ monomer (without and with T_4_) needed additional drying steps before performing measurements, due to the paste-like consistency caused by the occlusion of solvents. The samples were annealed at 70 °C for 2 h, 120 °C for 4 h, and 200 °C for 4 h in an oxygen atmosphere, after which a solid was obtained.

### 2.3. Annealing of Silicon Oxycarbide Samples

The annealing was performed in a tube furnace in an argon atmosphere. In order to remove all traces of air from the furnace, as well as excess solvent from the samples, the heating process consisted of four steps with set temperatures kept for the following times: 70 °C for 2 h, 120 °C for 4 h, 200 °C for 4 h, and the final annealing temperature for 30 min. The final annealing temperatures were 600, 700, 800 and 900 °C. The heating speed was 10 °C/min, and heating time was not taken into account in the previously mentioned steps. The furnace was cooled passively to room temperature. At 200 °C, the argon flow was closed and the furnace was opened.

### 2.4. T_4_M_2.67_^H^ Synthesis Progress and Product Structure Investigation

Intermediate-product structures of T_4_M_2.67_^H^ synthesis were investigated using medium infrared (MIR) spectroscopy. A BioRad FTS 3000 Excalibur spectrophotometer equipped with Miracle ATR module (Pike Technologies) with a zinc selenide crystal was used. The measurements were conducted in the 4000–550 cm^−1^ range, with 4 cm^−1^ resolution and 45° geometry. The final results were the accumulation of 32 scans.

The progress of T_4_M_2.67_^H^ synthesis (chlorodimethylsilane addition) was monitored using Shimadzu GC2010 gas chromatograph equipped with a thermal conductivity detector (TCD) and Zebron (0.25 mm diameter, 30 m length, polydimethylsiloxane as stationary phase). Helium was used as a mobile phase, with 1.53 mL/min flow speed. The column temperature was set to 60 °C for 4 min, then it was heated to 240 °C with 18 °C/min heating speed. The detector temperature was 250 °C.

The structure of T_4_M_2.67_^H^ was investigated using nuclear magnetic resonance. The ^29^Si NMR experiment was performed using a Bruker Advance III HD (400 MHz) spectrometer. The sample was dissolved in CDCl_3_.

### 2.5. Precursors and Silicon Oxycarbide Structure Investigation

The XRD experiment was conducted using the X-ray diffractometer X’Pert Pro MD (Philips). It employed Cu Kα X-ray lines with the Bragg–Brentano standard setup with a Ge (111) Johansson monochromator at the incidence beam. The scanning range was 5–65°, with 0.016° step size and 1 s measurement time for each step. The samples were spun along an axis parallel to the scattering vector during the measurements, to negate the effects associated with the development of a preferred orientation. The measurements were performed at room temperature.

The structure of the precursors and obtained silicon oxycarbide samples were investigated using middle infrared spectroscopy. The experiments were conducted using a Bruker VERTEX 70v spectrometer. The standard KBr pellet method was used, and measurements were taken in a vacuum. The one beam method with KBr pellet baseline subtraction was utilized. The reference KBr pellets were prepared prior to each experiment, using the same method as pellets containing samples in order to include water adsorbed by KBr in baseline correction. The measured range was 4000–400 cm^−1^, with 4 cm^−1^ resolution, and an accumulation of 128 scans gave the final spectra.

The thermogravimetric experiment was conducted using a Hi-Res TGA 2950 thermogravimetric analyzer (TA Instruments). The measurements were performed with a 10 K min^−1^ heating rate in an argon atmosphere.

An elemental analyzer CHNS (Vario Micro Cube) with electronic microbalance was used to perform elemental analysis of samples. The materials of the same type obtained through different syntheses and annealing processes were ground and analyzed. The obtained information included carbon and hydrogen atom content. The silicon atom content was calculated under the assumption that all the leftover material was SiO_2_. Even though it is theoretically possible to calculate molecular formula using this method, the discrepancies between different results for the same sample (e.g., caused by slightly different trace amounts of oxygen in the furnace or differences in the material between their surface and bulk) resulted in errors that made these formulas unreliable, while the carbon to silicon atom ratio error was comparatively minor.

## 3. Results

### 3.1. T_4_M_2.67_^H^ Synthesis and Structure

**Figure 2:**
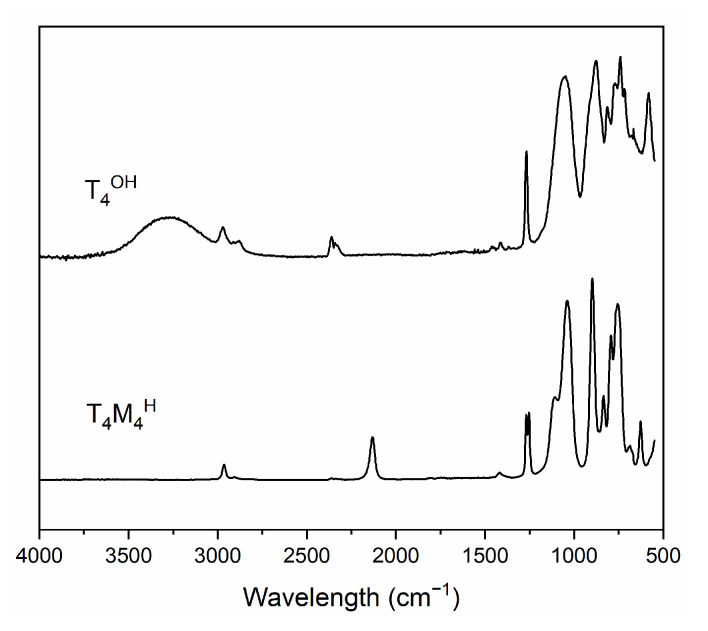
IR spectra of intermediate products in monomer synthesis.

The steps of T_4_M_2.67_^H^ synthesis were investigated using MIR (Figure 2).

In the case of both spectra, a number of bands typical for siloxanes and silsesquioxanes can be observed. These include stretching vibrations of C-H bonds at 2900–3000 cm^−1^ [25,31,32,33,34,35,36,37,38,39,40,41], bending vibrations of Si-CH_3_ groups at about 1270 cm^−1^ [25,31,38,39,40], and a number of bands that can be attributed to symmetric bending of Si-O-Si bridges between 790 and 900 cm^−1^ [38,39,40] and Si-O ring with 4 Si atoms bending vibrations at 565–585 cm^−1^ [39]. The Si-C bond stretching vibrations band can be found at 825–845 cm^−1^, its intensity enhanced by the coupling with C-H bond vibrations [38]. Vibrations of parts important for the reaction progress investigation are Si-OH stretching vibrations at about 3500–3600 cm^−1^ [25,31,41,42], which are very weak and can only be found in the case of the T_4_^OH^ sample and, most importantly, Si-H bond stretching vibrations at about 2130 cm^−1^ [36,38,39,41,43,44], which are only visible in the case of the T_4_M_2.67_^H^ sample. Other differences between spectra can be found between 1000 and 1200 cm^−1^, where stretching asymmetric vibrations of Si-O-Si can be found [31,38,39,40,41,42,45,46,47]. The T_4_^OH^ sample has one maximum in that region, while the T_4_M_2.67_^H^ sample has two. Bands found in the T_4_^OH^ sample spectra between 3000 and 3500 cm^−1^ can be attributed to O-H stretching vibrations [25,39,41], and at about 2350 cm^−1^ can be attributed to stretching vibrations in CO_2_ molecules [48]. From these spectra, it can be concluded that both reactions were conducted with nearly 100% efficiency, as bands that can be attributed to vibrations of reactive groups of substrates (namely Si-H and Si-OH) are no longer visible, and bands attributed to the product specific groups (namely Si-OH and Si-H) are present. Additionally, bands at 1000–1200 cm^−1^ show more a complicated structure of the Si-O-Si backbone in case the of the T_4_M_2,67_^H^ sample.

**Figure 3:**
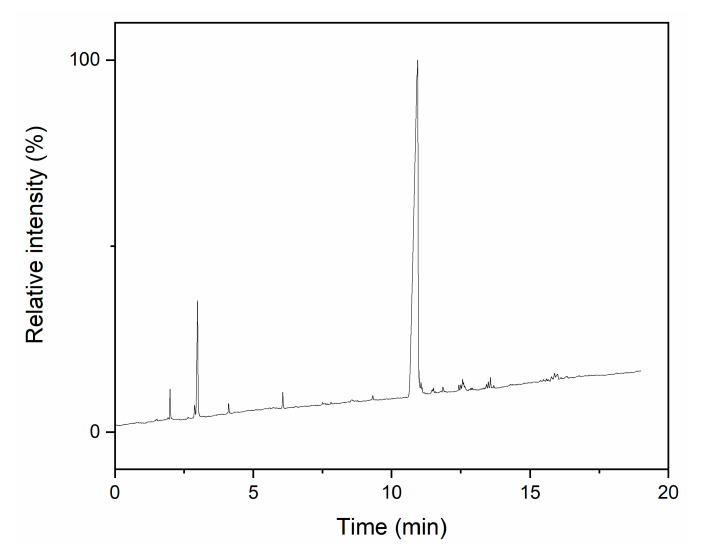
GC chromatogram of final product from initial synthesis.

**Figure 4:**
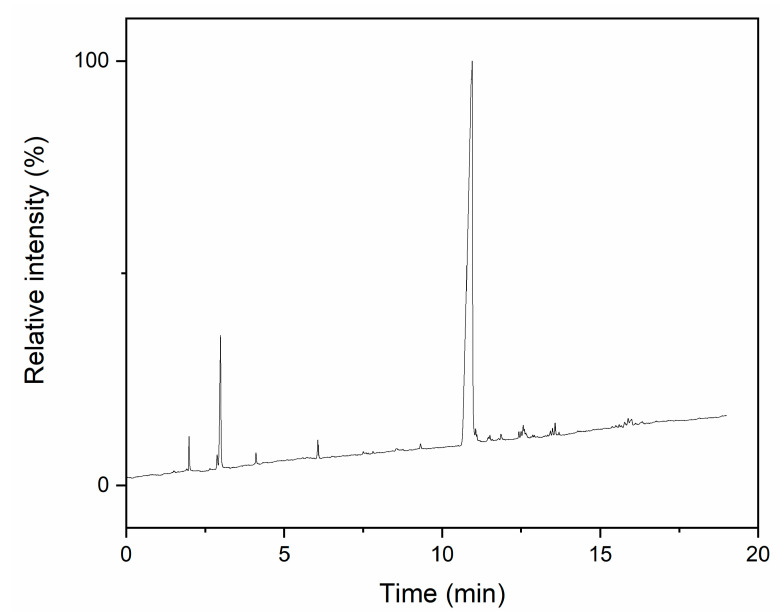
^29^Si NMR spectrum of T_4_M_2.67_ compound.

The structure and purity of the final product from the initial synthesis was investigated using GC (Figure 3) and ^29^Si NMR (Figure 4).

The GC chromatogram of synthesis products shows a number of weak signals between 2 and 6 min, which can be attributed to substrates. The most prominent one at 3.1 min shows that a large amount of pyridine was still present in the product after the purification process. This should not have had a large impact on further syntheses, as hydrogen substitution is very fast and polycondensation uses relatively large amounts of hydrochloric acid as a catalyst. Signals registered after 10 min can be attributed to products, with one clearly dominant, which shows that a single product was obtained.

The ^29^Si NMR experiment showed that, due to the extreme reactivity of Si-OH groups, even trace amounts of water molecules were able to cause oligomerization of T_4_^OH^ molecules. The band at −5.70 ppm can be attributed to M^H^ structural units, while bands at 64.90 and 66.31–66.61 ppm can be attributed to T structural units connected to a single M unit and connected only to other T units, respectively [38,49,50]. As such, from the band intensity, the 3:2 ratio of T and D (M^H^ before Si-H group substitution) structural units can be calculated. From the results of the GC experiment, one can conclude that the main product of these reactions the T_12_M_8_^H^ molecule, which, due to the use of T_4_ units as a base for the materials in this article, is referred as T_4_M_2.67_^H^.

### 3.2. Structure of Silicon Oxycarbide Materials and Their Precursors

**Figure 5:**
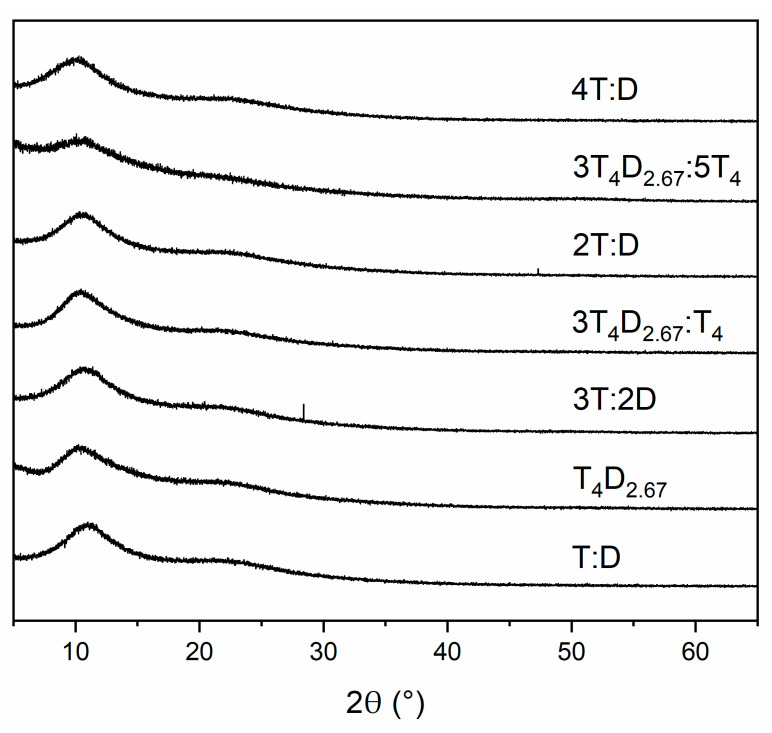
XRD analysis of precursors.

The XRD analysis of all the precursors (Figure 5) shows that all materials were amorphous. The two visible peaks at 10.1–11.0° and 21.5–22.7° are typical for systems based on T units and indicate a pseudo-ladder structure, with the first one corresponding to 8.0–8.8 Å distance between T_4_ rings building the bulk and the second one corresponding to 3.9–4.1 Å distance between grades in the T-based ladder [25,39,50]. The intensities of these peaks are similar regardless of whether the T_4_ rings were introduced in the monomers or not. The only difference is observable in the case of the 3T_4_D_2.67_:5T_4_ sample, where these peaks are less visible. This could be caused by more random conformation of T_4_ rings caused by dissimilarities in monomer structures and a similar molar amount of structural units in these monomers in this mixture, which leads to most random structures.

**Figure 6:**
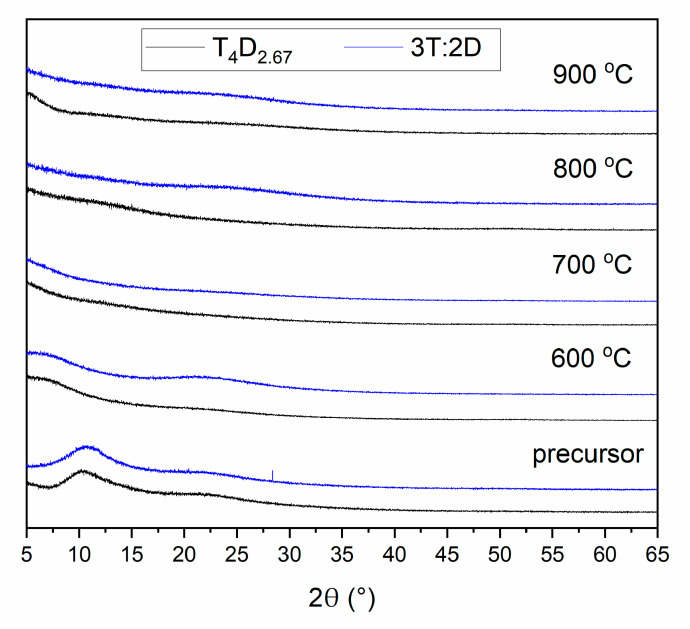
XRD analysis of materials obtained from precursors with 3:2 T:D ratio (T_4_D_2.67_ and 3T:2D) after annealing at temperatures between 600 and 900 °C.

The XRD analysis of samples with 3:2 T:D ratio (T_4_D_2.67_ and 3T:2D) obtained in 600, 700, 800 and 900 °C (Figure 6) shows a completely amorphous structure, with traces of precursor structure left after annealing at 600 °C. All other materials showed similar results. This confirms that 900 °C is too a low temperature to observe phase separation regardless of the monomers used.

**Figure 7:**
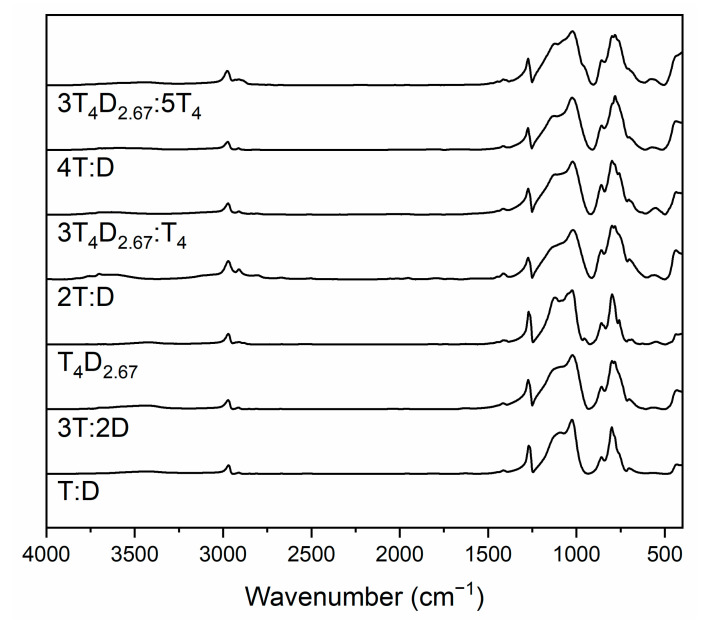
IR spectra of precursors.

The IR spectra of precursors (Figure 7) are similar to each other and share most bands with these found in the T_4_D_2.67_ monomer. This includes bands correlating to stretching asymmetric vibrations of Si-O-Si bridges between 1000 and 1225 cm^−1^ [31,38,39,40,41,42,45,46,47], especially ladder structure vibrations at about 1205 cm^−1^ [25,39], as well as O-Si-O structures at 455–470 cm^−1^ [38,39,43,45,46], and, most importantly, rings containing 4 Si atoms at about 565 cm^−1^ [39]. Si-C bond stretching vibrations band can be seen at 825–845 cm^−1^ [38] and complex Si-C-Hx bending vibrations for T and D units can be found at 760–780 cm^−1^ and 800 cm^−1^, respectively [51]. The main difference between spectra lies in bands correlated to C-H stretching vibrations at 2850–3000 cm^−1^ [25,31,32,33,34,35,36,37,38,39,40,41] and Si-CH_3_ bending vibrations at 1270–1285 cm^−1^ [25,31,32,33,34,36,37,38,39,40,41] that are a result of different D unit amounts between samples. The barely visible bands at about 3500 cm^−1^ can be attributed to unreacted Si-OH groups [25,31,41,42] due to sterical limitations, while the broad band between 3000 and 3550 cm^−1^ can be correlated to OH stretching vibrations in ethanol (~3430 cm^−1^)/water (~3380 cm^−1^) occluded in the polycondensed lattice and not removed completely by drying [25,39,41].

**Figure 8:**
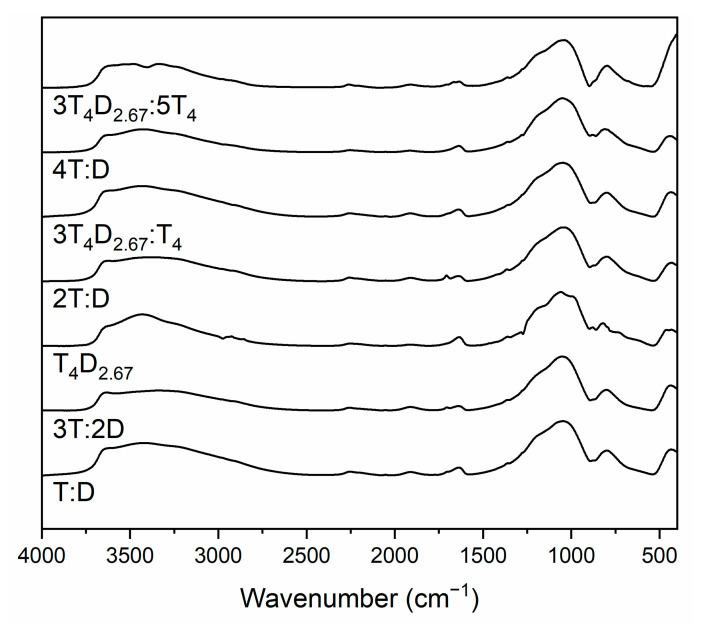
IR spectra of silicon-oxycarbide-based black glasses obtained from precursors after annealing at 800 °C.

IR spectra of silicon-oxycarbide-based black glasses obtained after annealing at 800 °C (Figure 8) consist of mostly the same bands as precursors, as the bands visible are mainly related to siloxane and the silsesquioxane lattice, which undergoes changes during the ceramization process, while retaining basic structural elements. Two additional bands at about 1710 cm^−1^ and 1600 cm^−1^ are correlated with stretching C=O bond vibrations [52] and bending vibrations in water molecules [25,39], respectively. The appearance of these bands in the spectra could be caused by hydrophilization of the material and subsequent adsorption of the water with some dissolved CO_2_ molecules on the surface of the ground material. This is confirmed by the great increase in the intensity of the band at about 3600 cm^−1^ attributed to Si-OH stretching vibrations, as well as the band at 3000–3550 cm^−1^, which in this case can be correlated with adsorbed water O-H stretching vibrations. This band’s intensity, when comparing pairs with the same initial Si:C ratio, shows greater hydrophilicity of the materials obtained from large precursors. This effect becomes less prominent with the decrease of the number of carbon atoms per silicon atom. All materials exhibit smooth a spectrum typical for glassy materials, with the exception of the T_4_D_2.67_ sample, where sharper bands and visible bands at 2850–3000 cm^−1^ attributed to stretching vibrations of C-H bonds indicate that the ceramization process had not been completed. The structure of the SiO_2_(C)-based lattice was very similar in all cases, with the exception of the 3T_4_D_2.67_:5T_4_ sample, in which case it is typical for materials obtained from the T_4_-monomer-based samples, while others are similar to materials obtained from simple monomers [28]. The amount of adsorbed water was also similar (due to the high surface charge of the ground powder, preventing precise weighting of samples, quantitative measurements were impossible), with the exception of the T:D and 3T_4_D_2.67_:5T_4_ samples, which adsorbed a significantly greater amount, which indicates the more hydrophilic character of these samples.

**Figure 9:**
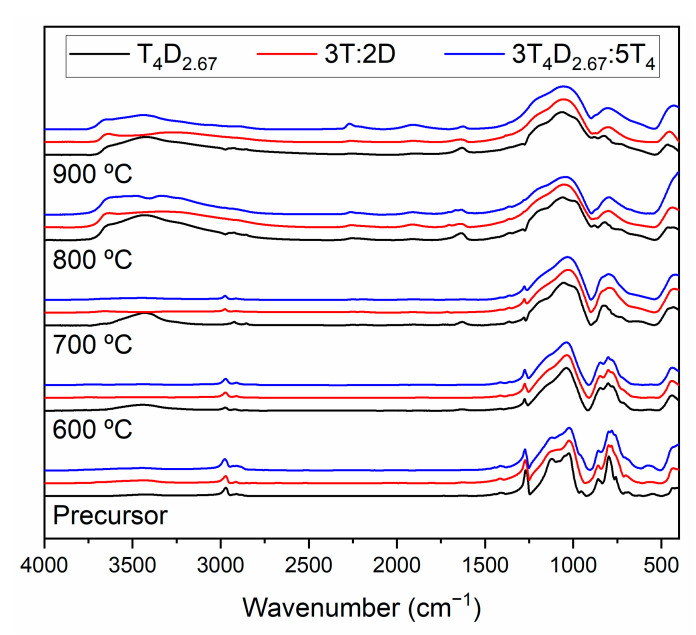
IR spectra of materials obtained at 600, 700, 800 and 900 °C from T_4_D_2.67_, 3T:2D and 3T_4_D_2.67_:5T_4_ precursors.

Based on thermal evolution (Figure 9), the precursors can be divided into two groups. In both cases there was a gradual smoothing of spectra features, indicating transition into glassy material from a polymeric one. The difference lies in hydrophilicity; large-monomer-based materials exhibited gradual change, which indicates the loss of organic groups either in the form of volatile hydrocarbons or the formation of a free-carbon phase throughout the annealing process, while simple-monomer-based materials had a distinct change between 700 and 800 °C, which can be connected to the formation of a free-carbon phase during the ceramization process. The 3T_4_D_2.67_:5T_4_ sample behaved similarly to the simple-monomer-based specimen in this regard, which can be attributed to the different distribution of carbon atoms in the bulk, probably due to large domains without D units, as these are fixed to T_4_D_2.67_ macromers. These studies also show the stability of the T_4_D_2.67_ sample structure, in which case the smooth spectrum of the glassy structure was not achieved even at 900 °C.

**Figure 10:**
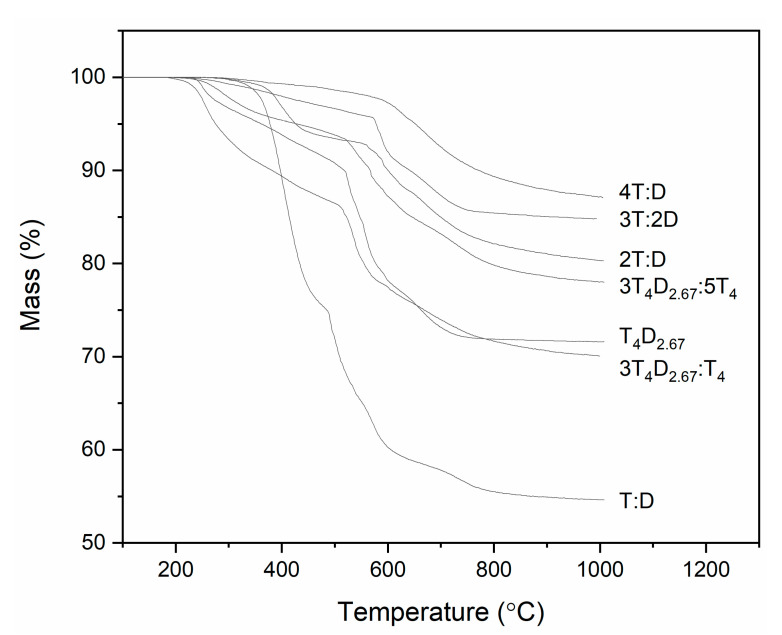
TG analysis of precursors.

The mass loss in the TG experiment (Figure 10 and Table 1) can firstly be attributed to the loss of occluded solvents, then to the sublimation of oligomers, then to the loss of hydrogen and carbon as molecules with very low molar mass, and finally to the ceramization process [27]. In general, in the case of small monomers, the amount of occluded solvents is minimal. In the case of larger molecules, while initially substantial, it can be removed by drying at 200 °C, and due to the preparation process, it is not shown in the TG experiment. The loss of volatile oligomers that occurs even up to 550 °C was most prominent, as expected, in the case of the T:D sample (25.06%), while in the case of the 4T:D sample, it was negligible (1.92%). In the case of large monomers, this step had the greatest impact on the 3T_4_D_2.67_:T_4_ sample (14.05%), while it was still substantial in the case of other the samples (9.90% for T_4_D_2.67_ and 6.07% for 3T_4_D_2.67_:5T_4_). This can be attributed to the need for a solvent, which causes long downtimes between monomers interacting with each other and the ability for macromonomers to fold and form bonds between its own reactive groups, preventing its incorporation into the silsesquioxane matrix, as well as the possibility of T_4_ monomer dimerization. The small addition of T_4_ monomers increases the chances of oligomer formation, while larger additions decrease it, as, due to the greater density of reactive groups, T_4_ monomers enable easier bulk formation. In the second step, the loss of hydrogen and carbon in the form of small organic molecules was similar in most cases (about 7%), except for T:D, T_4_D_2.67_ and 3T_4_D_2.67_:5T_4_. In the case of the T:D sample, it can be attributed to the formation of D units rich in nanodomains. For samples including macromonomers, where D units have a forced even distribution, the likely reason is the formation of pendant D units. These units, due to their positioning inside the precursor, were unable to form a second bond. This effect had the greatest impact on the T_4_D_2.67_ sample (13.89%), as most monomers here only had eight reactive groups for the 20 structural units. The small addition of T_4_ decreased the loss to 8.82%, as these monomers can act as linkers. The larger inclusion of T_4_ monomers reversed the process (a loss of 10.85%), as the possibility of T_4_-based oligomer creation may sterically hinder the reactions of D units in macromonomers. In the last step, namely ceramization, the loss was similar for all samples (about 4.5%).

Elemental analysis was used to determine the silicon to carbon atom ratio in the obtained materials after annealing at 800 °C (Table 2). Additionally, for the samples T_4_D_2.67_ and 3T_4_D_2.67_:T_4_ and their equivalents, the measurements were also conducted for samples obtained at 700 °C in order to discern the cause for carbon loss when macromonomers were used. The results show that, despite initial discrepancies in the amount of carbon atoms in the material, the amount of carbon atoms in silicon oxycarbide stayed at the same level of 0.64–0.69 carbon atoms per silicon atom. Different results were obtained for the samples 4T:D and T_4_D_2.67_, with 0.94 ± 0.15 and 0.74 ± 0.21 carbon atoms per silicon atom, respectively. In the case of the 4T:D sample, the low share of D units prevented their evaporation in the form of oligomers, while the dispersion of carbon atoms prevented the loss of carbon in the form of compounds with low molecular weight. In the case of the T_4_D_2.67_ sample, the lower loss was caused by the dispersion of D units, as well as their fixation to the macromonomer. Even though oligomer evaporation was observed, it was caused by folded macromonomer evaporation, thus was not affecting the silicon to carbon atom ratio. The materials obtained at 700 °C show that in the case of samples based on macromonomers, the loss in the last stage of annealing was small, contrary to the samples obtained using small monomers. The initial lowering of the carbon atom amount in the 3T_4_D_2.67_:T_4_ sample was most probably caused by the evaporation of mainly folded macromonomers, as the T_4_ amount was too small to reliably create dimers.

The discrepancies between the results found in other articles [25,31] and for materials obtained from precursors based on small monomers described in this article are caused by the difference in sol-gel methodology. In the case of prior research, both the pressure and ambient humidity were kept constant as open vessels were used. In the case of this research, the use of closed vessels resulted in a gradual change of humidity in the experimental setup, from zero to saturation value, which in turn changed the speed of polycondensation, until it was suspended due to the inability to remove the reaction side product—water. The reaction was then restarted with an accelerated rate when sol was placed in the exiccator.

## 4. Discussion

From the aforementioned results, a number of conclusions can be drawn. Even though the structures of precursors obtained from macromonomers containing a number of D and T units and those containing a mixture of simple D and T monomers look similar at first glance, there is a significant difference in the thermal evolution of the materials, which results in materials of different structure. The predetermined bond between D and T units have a twofold effect—the loss of D units in the form of oligomers is mitigated, as the loss of whole macromonomers occurs, but the existence of pendant D units is made possible due to low density of reactive groups. The pendant unit’s methyl groups or whole pendant unit loss is the most probable reason for carbon atom loss at temperatures below 700 °C, as was shown in case of T_4_D_2.67_ and derivative samples. The forced carbon atom distribution also has a large impact on the silicon oxycarbide structure. The lack of distinctive D or T unit domains decreases the loss of carbon atoms in the ceramization process, but on the other hand, promotes a free-carbon phase formation. This can be inferred from the increased intensity of water related bands in the IR spectrum of samples prepared using macromonomers. This indicates the removal of carbon atoms from the ceramic bulk, even though these samples contain more carbon, as seen in the elemental analysis experiment. This effect can only be seen in precursors containing D units (which act as free-carbon phase formation initiators), as in the case of materials created using only T units (with the exception of the sample where D units were formed due to additional reactions), a better carbon atom dispersion promotes the formation of silicon oxycarbide bulk [28]. The stability of the macromonomer-based precursor (the T_4_D_2.67_ sample), in comparison with the two macromonomer mixtures, could be caused by the lower amount of unreacted active groups, as relative positioning can greatly impact the number of interconnections between rigid T_4_ monomers. On the other hand, in the case of simple monomers, the high probability of methyl group nanodomain formation can cause an easier free-carbon phase formation initiation, thus making them undergo ceramization at lower temperatures than macromonomer-based precursors [28].

## Figures and Tables

**Table 1:** Mass loss in each annealing step.

	Annealing Step		
Sample	I	II	III
T:D	25.06%	15.74%	4.38%
T_4_D_2.67_	9.90%	13.89%	4.49%
3T:2D	4.06%	6.90%	4.09%
3T_4_D_2.67_:T_4_	14.05%	8.82%	5.49%
2T:D	6.41%	6.75%	6.21%
3T_4_D_2.67_:5T_4_	6.07%	10.85%	4.89%
4T:D	1.92%	7.03%	3.69%

**Table 2:** Carbon atoms per silicon atom in materials after annealing.

Sample	Carbon Atoms per Silicon Atom in the Material		
	Theoretical	Experimental	
		700 °C	800 °C
T:D	1.50	-	0.64 ± 0.04
T_4_D_2.67_	1.40	0.82 ± 0.05	0.74 ± 0.21
3T:2D	1.40	0.85 ± 0.05	0.68 ± 0.02
3T_4_D_2.67_:T_4_	1.33	0.70 ± 0.22	0.64 ± 0.03
2T:D	1.33	0.86 ± 0.18	0.69 ± 0.06
3T_4_D_2.67_:5T_4_	1.20	-	0.69 ± 0.03
4T:D	1.20	-	0.94 ± 0.15

## Data Availability

Not applicable.
